# Combined effects of virtual reality techniques and motor imagery on balance, motor function and activities of daily living in patients with Parkinson’s disease: a randomized controlled trial

**DOI:** 10.1186/s12877-022-03035-1

**Published:** 2022-04-30

**Authors:** Muhammad Kashif, Ashfaq Ahmad, Muhammad Ali Mohseni Bandpei, Syed Amir Gilani, Asif Hanif, Humaira Iram

**Affiliations:** 1 University Institute of Physical Therapy, Faculty of Allied Health Sciences, University of Lahore, 1 KM Defence Road, Lahore, 4200 Pakistan; 2 Riphah College of Rehabilitation and Allied Health Sciences, Riphah International University, Faisalabad Campus, Faisalabad, Pakistan

**Keywords:** Parkinson’s disease, Virtual reliability, Motor imagery, Physical therapy, Rehabilitation

## Abstract

**Background:**

Parkinson's disease (PD) is the second most prevalent neurodegenerative disorder, impairing balance and motor function. Virtual reality (VR) and motor imagery (MI) are emerging techniques for rehabilitating people with PD. VR and MI combination have not been studied in PD patients. This study was conducted to investigate the combined effects of VR and MI techniques on the balance, motor function, and activities of daily living (ADLs) of patients with PD.

**Methods:**

This study was a single-centered, two-armed, parallel-designed randomized controlled trial. A total of 44 patients of either gender who had idiopathic PD were randomly allocated into two groups using lottery methods. Both groups received Physical therapy (PT) treatment, while the experimental group (N: 20) received VR and MI in addition to PT. Both groups received assigned treatment for three days a week on alternate days for 12 weeks. The Unified Parkinson’s Disease Rating Scale (UPDRS) (parts II and III), Berg Balance Scale (BBS), and Activities-specific Balance Confidence (ABC) Scale were used as outcome measures for motor function, balance, and ADLs. The baseline, 6^th^, and 12^th^ weeks of treatment were assessed, with a 16^th^ week follow-up to measure retention. The data was analysed using SPSS 24.

**Results:**

The experimental group showed significant improvement in motor function than the control group on the UPDRS part III, with 32.45±3.98 vs. 31.86±4.62 before and 15.05±7.16 vs. 25.52±7.36 at 12-weeks, and a *p*-value < 0.001. At 12 weeks, the experimental group's BBS scores improved from 38.95±3.23 to 51.36±2.83, with *p*-value < 0.001. At 12 weeks, the experimental group's balance confidence improved considerably, from 59.26±5.87to 81.01±6.14, with a *p*-value of < 0.001. The experimental group's ADL scores improved as well, going from 22.00±4.64 to 13.07±4.005 after 12 weeks, with a *p*-value of < 0.001.

**Conclusion:**

VR with MI techniques in addition to routine PT significantly improved motor function, balance, and ADLs in PD patients compared to PT alone.

**Trial registration:**

IRCT20200221046567N1. Date of registration: 01/04/2020

## Introduction

Parkinson’s disease (PD) is the second most common neurodegenerative disorder. It is characterized by the degeneration of dopaminergic neurons and an accumulation of Lewy bodies in the midbrain; however, as the disease develops, the spinal structures, limbic system, forebrain, and neocortex are also affected [1]. The neurodegenerative process underlying PD has not yet slowed down or stopped. A typical PD patient is usually represented as an elderly man with bradykinesia, tremor at rest, and impaired gait [2].

Physical therapy (PT) is management opportunity available for maximizing functionality using movement rehabilitation in patients with PD, with a focus on upper extremity functioning, maintaining posture, enhancing balance, improving gait, transfers, and augmenting physical activity. Various techniques have been suggested in PT, including routine PT, treadmill walking, cueing, dancing, or any martial arts depicting temporary improvements in gait speed, freezing of gait, balance, motor skills, fall risks, activities of daily living (ADLs), and quality of life [3]. However, few studies looking into the effects of PT treatment in its various forms have reported a loss of exercise benefit within weeks or months of cessation of exercise protocol [4, 5]. In addition, several barriers to exercise compliance due to fear of falling, longer treatment duration, financial pressure, patient safety, and lack of time have been observed in PD patients [4].

The use of virtual reality (VR) has emerged as a favorable rehabilitation choice for PD, as it has the potential to improve long-term exercise compliance in a tailored, engaging, and motivational way. VR increases the chances of regaining lost movement skills because it enhances the movement and cognitive processes of its participants [6]. VR techniques stimulate movements, optimize motor learning pathways while compensating for non-functional neural networks in collaboration with external sensory inputs, and empower the external feedback system [7]. Bringing a challenging, inspiring, and motivating environment to envision motor training, the playful mechanism of a VR system enables patients to perform exercises with increased frequency according to individual needs. The literature has reported many feasibility studies incorporating gaming systems into rehabilitation and those available commercially, such as Nintendo Wii ™ [8], exergaming [9], Gamepad system [10], home-based Nintendo Wii Fit system [11], balance-based exergaming [12] VR Wii [13], and a custom-made VR system for managing PD patients [14]. Good feasibility has been reported for these devices to be used for balance training and activities pertaining to movement, mood, and ADLs.

Motor imagery (MI) is the imaginary execution of motor activities or the activation of specific muscles when there is an absence of any sort of explicit feedback [15]. The efficacy of this domain of rehabilitation has been shown to improve and develop motor skills in many neurological pathologies in which the patient presents with motor recognition and execution impairments [16]. MI can be implemented at all stages of recovery from PD, is very effective in movement-related pathologies, and can be performed self-sufficiently [16, 17]. MI is a key option for rehabilitation because this technique has a minimal risk of physical injury, a high level of accessibility, ease of availability, minimal financial cost, and minimal need for equipment. Moreover, this innovative technique based on explicit learning can target various motor and non-motor aspects of one’s performance [18–20]. Note that in the advanced stages of PD, when physical activity becomes intensively limited, MI is the recommended relevant technique. It can be customized according to individual needs, be used in group form (i.e., the patients can be engaged either physically or virtually), and be used for underserved patients, making it highly relevant for PD patients [21–23]. During MI, areas related to motor perception, the premotor cortex, and the lower parietal lobule are stimulated. This happens with normal movement implementation and is related to core motor learning mechanisms without execution. However, the level of imagination and motor performance depends on the complexity of the tasks [24].

The presence of PD in our society, the limited time available for patient management, the costly outpatient therapy, and the increased use of expensive electro-therapeutic equipment have led members of the rehabilitation team to develop sources that can design innovative, alternative, and time-efficient methodologies, such as the use of VR therapy and MI techniques. Furthermore, both techniques have been shown to be beneficial in improving balance and motor function in patients with various of neurological conditions. VR has been demonstrated to help enhance physical function in traumatic brain injury [25], vestibular rehabilitation [26], stroke [27] and cerebral palsy [28]. There is ample evidence that MI practice improves motor performance and learning in people with neurological conditions such as multiple sclerosis, stroke, spinal cord injury [29].

There is a vital need to conduct research on the identification of new treatments for people with PD. VR and MI are innovative therapeutic techniques to improve the balance and mobility of people with PD. These techniques can improve compliance by encouraging patients to perform exercises in a motivating, entertaining, and engaging manner. The MI process relies on explicit learning, whereas the VR process relies on implicit learning. These learning techniques can be used in alongside to advance learning that enhances balance and motor function in people with neurological conditions. However, the evidence for synergic use of MI or VR in conjunction with routine PT is currently lacking [30]. In addition, to the best of the authors’ knowledge, no study has combined MI with VR as a complementary technique to improve motor learning. In practice, although the first choice is always considered to be the implementation of PT treatment for PD patients, it requires long treatment duration to produce effects; therefore, patient compliance is always an issue. This unique randomized controlled trial study aimed to investigate the comparative effects of VR with MI techniques, in addition to routine PT and routine PT alone, on balance, motor function, and ADLs in PD patients.

## Methods

### Study design

This clinical trial with two-armed, parallel- design was conducted in the Department of Physical Therapy, Safi Hospital, Faisalabad, Pakistan in 2021. This was a single blinded study in which only the assessor was blinded. Due to the nature of the intervention, patients and the principal investigator could not be blinded. Moreover, the statistician was also kept blinded from the group allocation of the patients by providing the data in anonymized form and was pre-coded before being handing him over.

### Study participants

Subjects with a diagnosis of PD were recruited from the neurology and neurosurgical departments of hospitals in Faisalabad. The patients were then referred to the Department of Physical Therapy, Safi Hospital, where they were further assessed by the physical therapist (who was also movement specialist) for their eligibility to be selected for the trial. Those aged 50–80 years with idiopathic PD, severity ranging from stage I to stage III on the modified H and Y scale, intact cognition according to their mini-mental score examination (MMSE) score (greater than or equal to 24) [31], and transfer independence were selected for participation in the study [32]. Patients with any other neurological presentation, orthopedic pathology, visual anomalies, cardiovascular issues, severe dyskinesia or “on–off” phases, a history of surgery for PD, a history of virtual games used for treatment in the last three months, and virtual game phobia were excluded from the study. Written informed consent was obtained from the participants before their participation in the study.

### Sample size calculation

The study sample size was calculated using the mean Unified Parkinson’s Disease Rating Scale (UPDRS) as 25.1 ± 12.8 and 18.5 ± 11.0 for the VR group and control group, respectively, with a confidence interval (α) of 95% and 80% power of the study extracted from Yang et al. [14]. To detect statistically significant differences, 28 patients were required. However, a 20% expected drop-out rate during the study period must be managed.

### Randomization

**Fig. 1:**
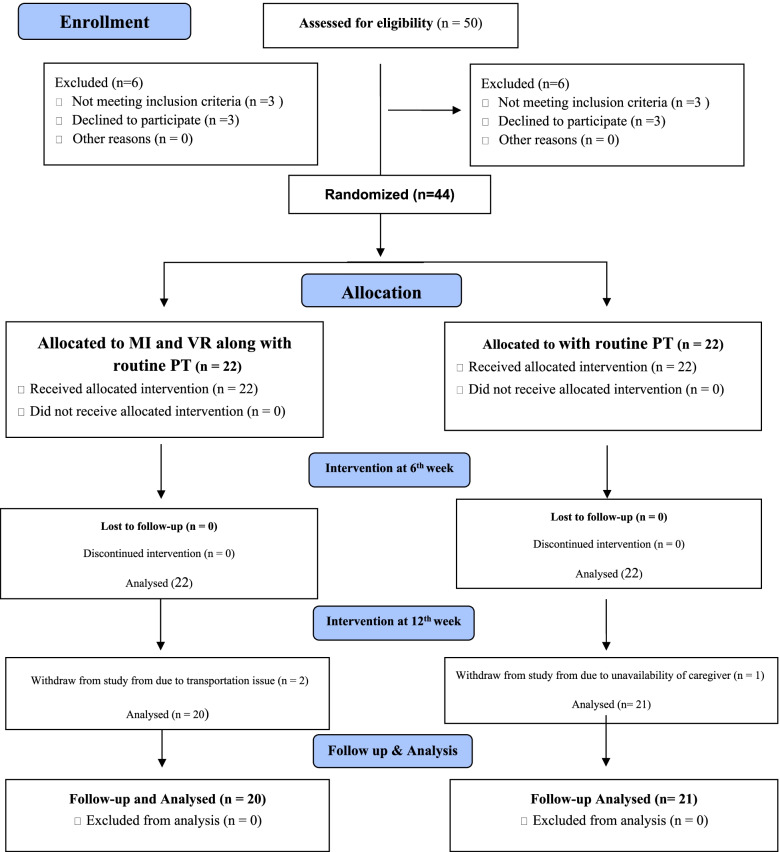
CONSORT study flow diagram

Followed by baseline assessment randomization was carried out by a random assignment according to a lottery procedure. Demographic variables were used as an input for the process of minimization. Each participant was assigned a number by the main auditor, and the numbers were then drawn at random from a box, forming a random sample. The participants’ ratio of 1:1 was maintained for the experimental and control groups during this trial. The CONSORT diagram for the study is presented in Fig. 1.

### Groups and intervention procedures

Voluntary written consent was given by the subjects, who were then randomly divided into two groups after signing the informed consent form for the study. Group A (control) received routine PT (including warm-up, stretching, strengthening, and relaxation exercises; limb coordination exercises; and core, neck, and gait training) (Routine PT only), whereas Group B (experimental) received routine PT protocols, along with VR and MI techniques (Routine PT+ VR+ MI). Each group consisted of 22 participants at the baseline. In group A, the patients received 40-min sessions and 20 min of walking and cycling, with a short rest period every other day (three days a week) for 12 weeks. The patients in Group B received 60-min sessions (including 40-min routine PT as in Group A, 10–15 min of VR, and 5–10 min of MI techniques) every other day (three days a week) for 12 weeks.

The subjects were evaluated by an independent assessor who was unaware of the study objectives and the group allocation of the subjects. At baseline, the participants were assessed for motor function using the UPDR-III, Berg Balance Scale (BBS) for balance, Activities-specific Balance Confidence (ABC) for balance confidence, and UPDRS-II for ADL’s. The intervention and assessments took place at the same time of day and in the ON medication state (2 hours after taking the medication) [33, 34]. Patients were assessed late in their ON phase because of the pharmacodynamics of levodopa (the onset of medication effect is 20–40 minutes and the duration of effect is 2–4 hours after medication) [35, 36]. Furthermore, the medication regimens of all study participants remained unchanged throughout the period of the study. Because of the potential of interference in the results of the study, patients with on-off motor fluctuation and dyskinesia above grade 3 on the UPDRS were excluded from the study [37].

### Interventions

The interventions used in both the experimental and control groups were based on a previously reported protocol for the rehabilitation of PD with VR and MI, in addition to routine PT treatment [32].

### VR rehabilitation protocol

**Fig. 2:**
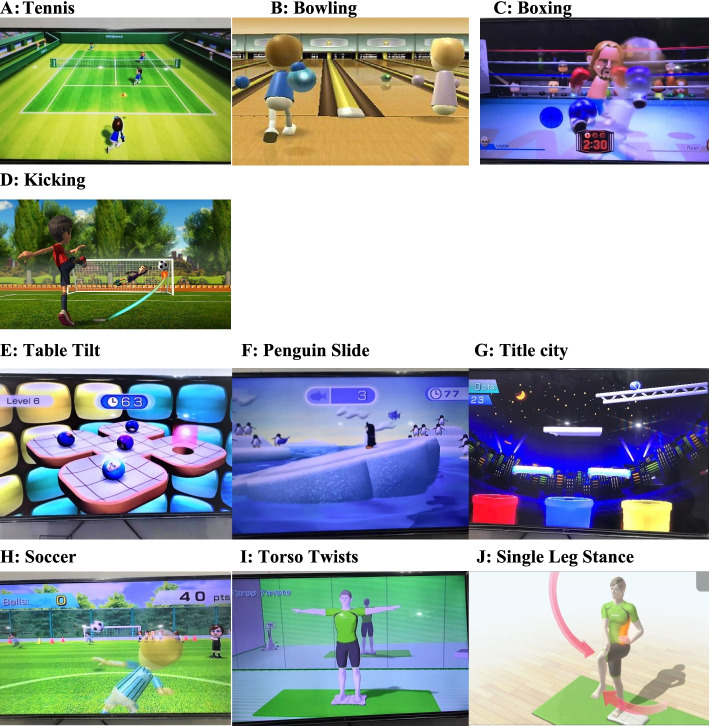
Different types of games used in VR training

The length of the VR application was 10–15 min during each session for each participant. The VR system consisted of a wall-mounted display, a Wii box, a Wii remote, and a Wii Fit board. The patients were instructed to stand on Wii Fit board while interacting with the VR system and playing the selected games. A panel of three senior physical therapists (movement specialists) selected the games for three domains—motor functionality, balance, and ADLs—based on a previously conducted systematic review [38]. Multiple options were available on the Wii box, varying from easy to increasing difficulty. As part of the treatment protocol, two sessions were given as practice sessions to familiarize the patients with the environment and the VR system and to develop a rapport between the therapist and the patients. The games, the significance of the therapy, and the scoring of each game were explained to the patients. For the sessions in the first three weeks, the selected games for improving motor function and balance were at an easy level. For this study, the games for improving motor function included tennis, boxing, bowling, and kicking, while the games for dynamic balance training included soccer, table tilt, penguin slide, tilt city and for static balance were single-leg extension, and torso twist [39–41] (Fig. 2).

For safety purposes, the patients stood inside parallel bars on the Wii Fit board with their shoes off. The therapist stood behind the patients for instructions, prompt feedback (when required), and monitoring of their performance. To start the VR session, balance games were played. Each exercise session included two games for dynamic balance and one for targeted improvements in static balance. Based on the level of difficulty, exercises were selected, and the difficulty level was gradually increased according to the patients’ performance. Starting from the penguin slide, they progressed to table tilt; tilt city, and, finally, soccer. Initially, each game was played for 2–3 min per session. With the progression of performance, 3–4 min of table tilt was added. While playing this game, a typical mobility pattern was initiated, and weight shifts improved with the activity. In the same week, the subjects performed single-leg extensions for 1–2 min. In the following weeks, tilt city, soccer, and torso twists were added to the individualized plan. The subjects performed these activities for 1–5 min per session. Treatment sessions then progressed to motor function games, including bowling, tennis, kicking, and boxing (least challenging to most challenging), with most treatment sessions ending with boxing. The subjects were able to perform most of the games with minimal guidance. Boxing was performed in the last three weeks of therapy because of increased balance and coordination demands [32].

### MI rehabilitation protocol

The last 5–10 min of the session comprised the MI, and a three-step process was used to incorporate the technique. As a first step, the participants were instructed to watch the recorded videos. Two categorical sets of videos were available: one with normal movements and the other containing recordings of patients performing the movements. The patients were instructed to watch and analyze the differences in both videos. In the next step, they were instructed to relax and concentrate on their quiet breathing patterns. Instructions were given to the participants for a comfortable and relaxed sitting posture while they were in the chair, with their back and arms supported. They were asked to close their eyes and perform slow nasal breathing while maintaining their focus. This was repeated 10 times. The patients were then asked to perform the activities, and verbal commands were given whenever needed. During the recall process, the components of the movements deviating from the normal were emphasized. The difficulty level of the activities and the analysis of the movement components increased gradually according to the patients’ capacity [32].

### Routine PT treatment

Each session began with routine PT treatment and lasted for 40 min in total. To begin, the patients were guided through warm-up exercises. While sitting comfortably on a chair with their backs and feet well supported, the patients were instructed to breathe in and out. Warm-up exercises were carried out for 5 min, with five repetitions of each exercise. The patients were guided through the execution of breathing properly, avoiding shallow breathing, straining, and holding their breath at any point during the session. They were asked to practice while lying supine on the bed and under the supervision of the principal investigator. Stretching exercises were performed for 15 min per session, and stretches were held for 10–30 s with four repetitions of each of the following areas: upper chest and neck flexors, shoulder flexors and adductors, elbow and wrist flexors, knee flexors, calves, and lower back. Strengthening exercises were also performed for 15 min during each session, with each exercise repeated 10–15 times. The following muscles were targeted for this training: core muscles (abdominals) and hip, knee, back, and elbow extensors. For cool-down, slow sustained stretches of shoulder flexors, adductors, and hip and knee flexors were performed for 5 min [32, 42].

### Adverse event records

An adverse event is any unfavorable medical event that occurs to a patient during or after treatment in a clinical study [32]. Nausea, dizziness and vertigo, are well-reported and common negative consequences of VR [43], also known as cyber or simulator sickness [44]. During the trial, all adverse events were considered.

### Outcome measures

A blind assessor recorded the scores obtained by the patients on UPDRS-part III, BBS, ABCS, and UPDRS-part II for motor function, balance, and ADLs at baseline, 6^th^, 12^th^, and 12^th^ week of therapy and during follow-up (16^th^ week), which was performed after one month of discontinuation of therapy. UPDRS is a renowned self-report and clinical observation tool frequently used to assess and monitor the progress of patients with PD for motor function and ADLs using different paradigms. Two subscales of UPDRS were used in this study: subscale II for rating ADLs and subscale III for rating motor function. Excellent internal consistency was found in many studies on UPDRS [45, 46]. Considering the rater consistency of the tool, it has been labeled as having adequate inter-rater reliability and intra-rater reliability for the section-II [47] and III [48, 49]. The BBS is the most commonly used assessment tool in combination with UPDRS in the clinical setting of rehabilitating PD patients, with high inter-rater and intra-rater reliability [50, 51]. The ABC scale is a self-administered scale used to predict balance confidence among patients with neurological issues. A score of 100% indicates full confidence, and 0% indicates no confidence in performing activities [52].

### Statistical analysis

The pre-specified primary null hypothesis for the analysis was that there was no difference in motor function measured with the UPDRS-III and balance measured by BBS between Routine PT only and Routine PT+ VR+ MI groups. The secondary null hypothesis was that there was no difference in ADL’s measured by UPDRS-II and balance confidence measured by ABCS between Routine PT only and Routine PT+ VR+ MI groups.

Data entry and statistical analysis were conducted using SPSS version 24. Descriptive analysis using mean, median, mode, variance, and standard deviation was performed for quantitative data, such as the participants’ age, gender, age of onset of PD, and PD diagnosis. The normality of the data was tested using the Kolmogorov–Smirnov and Shapiro–Wilk tests. The normality assumptions were not followed, as the data of the control and experimental groups did not follow a normal distribution. Thus, the Mann–Whitney U test was used for comparison. To determine which intervention was effective, the changes in the mean scores were analyzed. The significance for the data was set to p < 0.05. Due to the loss to follow-up at the time of data analysis, the number of participants was 20 in the experimental group and 21 in the control group. For motor function, balance, and ADLs, a post hoc responder analysis was used to assess clinical significance. It is recommended that the clinician determine whether a reported statistically significant difference translates to a meaningful clinical benefit, a change that the patient or the clinician would consider important for the patient's health or overall quality of life, this is referred to as clinically important important difference (CID), and the lower threshold limit of this measure is the minimum clinically important difference score (MCIDs) [53, 54]. The CID scores of UPDRS-II and III were based on the study by Shulman et al. [55] while for BBS reference values were used as per the study conducted by Chen et al. [56].

## Results

At the baseline, 44 participants were found to be eligible for the study because they met the inclusion criteria. All of these participants completed the 6-week assessment. Few of them dropped out at the 12-week assessment (2 from the experimental group and 1 from the control group) owing to transportation concerns or caregiver availability, therefore the final number of participants at the 12 and 16-week assessment was 20 in the experimental group and 21 in the control group. Both groups had comparable baseline data for age (years), PD duration, onset age, PD diagnosis, Hoehn-Yahr stage mean, and MMSE. The experimental group's mean age (years) was 63.86± 4.57, whereas the control group's was 62.31± 4.61. The experimental group had a mean age of onset of PD of 56.00 ± 4.06 vs 55.50± 4.06. The experimental group's mean age at PD diagnosis was 59.55± 3.91, whereas the control group's was 60.044.13. Means duration of disease was 6.23± 1.85 years in the experimental group and 6.55 ± 1.68 years in the control group. The experimental group's mean H&Y stage was 2.11 ± 0.74, whereas the control group's was 2.25 ± 0.67. The experimental group's mean MMSE was 26.41± 1.91, whereas the control group's was 27.29± 4.38 (Table 1).

**Table 1:** Demographic information of the participants

	Randomized (*n*=44)		*p*-value	Lost to post-test follow-up at 12^th^ week (*n*=3)	
Variables	Experimental Group	Control Group		Experimental Group	Control Group
	(*n*=22)	(*n*=22)		(*n*=2)	(*n*=1)
Age (years)	63.86±4.57	62.32±4.61	.936	61.00±2.83	60
Gender					
Female	9 (41%)	10 (45.45%)		01	00
Male	13 (59%)	12 (54.55%)		01	01
Height (cm)	160.36 ± 3.70	164.36 ± 2.68	.397	163.0 ± 0.0	161
Weight (kg)	59.59 ± 4.90	60.73 ± 5.43	.710	56.00±1.41	70
Disease duration (years)	6.23±1.85	6.55±1.68	.887	4.00±2.83	6.0
Age at onset of PD	56.00±4.06	55.50±4.53	.912	54.50±2.12	54
Age at diagnosis PD	59.55±3.91	60.05±4.13	.443	57.50±0.71	58
H&Y Stage	2.11 ± 0.74	2.25 ± 0.67	.720	2 ± 0.0	3.0
MMSE	26.41±1.91	25.27±4.38	.029	27.01±0.0	27.0

*PD* Parkinson’s disease, *MMSE* Mini mental state examination, *H&Y* Hoehn and Yahr Stage

### Between group differences

Motor function was assessed using the UPDRS-III. The mean score on UPDRS-III at baseline was 32.45±3.98, which changed to 23.00±8.31 after 6-weeks then decreased to 15.05±7.16 after 12 weeks of therapy and at follow-up was found to be 18.68±7.04, in the experimental group. While the mean score on UPDRS-III decreased from 31.86±4.62 to 28.23±6.10 after 6-weeks, then to 25.52±7.36 after 12 weeks and at 16-weeks was recorded to be 24.33±9.53 in the control group. Thus, significant differences were observed in the experimental group, with a *p*-value=.032 at 6-weeks, *p*-value<.001 at 12 weeks and *p*-value=.021 at 16-weeks respectively. The BBS scores on balance improved from 38.95±3.23 to 46.59±3.07, 51.36±2.83 and 52.36±2.30 in the experimental group at 6, 12 and 16-weeks respectively and from 40.23±4.61 to 43.23±4.45, 45.77±4.52 and 45.54±3.98 in the control group at 6, 12 and 16-weeks respectively. In the intervention group, the balance confidence of the patient improved significantly, with the scores changing from 59.26±5.87 to 73.55±4.45, 81.01±6.14 and 78.60±5.76 at 6, 12 and 16-weeks respectively, with respective *p*-values < 0.05. The ADLs also showed improvement in the intervention group, with scores varying on UPDRS-II from 22.00±4.64 to 17.14±4.36, 13.07±4.005 and 12.85±4.050 at 6, 12 and 16-weeks respectively. A *p*-value < 0.05 showed statistically significant differences (Table 2).

**Table 2:** Difference between groups regarding the mean scores of UPDRS-part II&III, BBS and ABCS

Outcome	Outcome Measures	Groups	Baseline	Assessment at 6^th^ Week	Assessment at 12^th^Week	Follow up at 16^th^ Week
			Mean ± SD	Mean± SD	Mean± SD	Mean± SD
Motor Function	UPDRS-part III	Experimental	32.45±3.98	23.00±8.31	15.05±7.16	18.68±7.04
		Control	31.86±4.62	28.23±6.10	25.52±7.36	24.27±9.23
		Z	-.329	-2.140	-4.146	-2.303
		*P*-value	.742	.032	<.001	.021
Balance confidence	ABCS	Experimental	59.26±5.87	73.55±4.45	81.01±6.14	78.60±5.76
		Control	59.34±8.89	66.39±7.80	71.83±8.25	68.65±7.33
		Z	-.282	-3.029	-3.240	-3.771
		*P*-value	.778	.002	<.001	<.001
Balance	BBS	Experimental	38.95±3.23	46.59±3.07	51.36±2.83	52.36±2.30
		Control	40.23±4.61	43.23±4.45	45.77±4.52	45.54±3.98
		Z	-1.084	-2.805	-3.953	-4.971
		*P*-value	.278	.005	<.001	<.001
Activities of Daily livings	UPDRS-part II	Experimental	22.00±4.64	17.14±4.36	13.07±4.005	12.85±4.050
		Control	21.51±3.89	20.03±3.80	18.00±4.19	16.54±4.61
		Z	-.166	-2.101	-3.583	-2.612
		*P*-value	.869	.036	<.001	.009

*UPDRS* Unified Parkinson’s Disease Rating Scale, *ABCS* Activities-specific Balance Confidence scale, *BBS* Berg Balance Scale

As the results revealed statistically significant differences between both study groups, in the next step post-hoc analysis was performed. This analysis was aimed to determine the clinical significance of the results. The following findings were obtained using the MCIDs for UPDRS-II, III, BBS, and ABCS. The experimental group improved from 68.2% at 6 weeks to 95.0 % at 12 weeks and follow-up evaluation using UPDRS-III. Among the control group, 36.4 percent showed improvement at 6 weeks, 57.1 percent at 12 weeks, and 61.9 percent at 24 weeks. Concerning the balance system, the intervention group exhibited clinically significant effects at follow-up, with over 90% of patients presenting with better balance, while only 47.6% of individuals improved balance on BBS. On the UPDRS-II, 90% of the study participants indicated independence in performing daily living activities in the intervention group, whereas only 10% improved in the control group. Finally, the values of ABCS demonstrated that 90% of the study participants in the intervention group had increased balance confidence (Table 3).

**Table 3:** Difference between groups in proportion of responders in outcome measures

Outcome	Outcome Measures	Groups	Assess-ment at 6^th^ Week	Assess-ment at 12^th^Week	*p*- value	Assess-ment at 6^th^ Week	Follow- up at 16^th^ Week	*p*- value	Assess-ment at 12^th^Week	Follow- up at 16^th^ Week	*p*- value
			n(%)	n(%)		n(%)	n(%)		n(%)	n(%)	
Motor Function	UPDRS-Part III	Exp.	15(68.2)	19(95.0)	.250	15(68.2)	19(95.0)	.250	19(95.0)	19(95.0)	.050
		Control	8(36.4)	12(57.1)	.005	8(36.4)	13(61.9)	.007	12(57.1)	13(61.9)	.032
Balance confidence	ABCS	Exp.	16(727)	17(85.0)	.009	16(727)	18(90.0)	.053	17(85.0)	18(90.0)	.016
		Control	6(27.3)	8(38.1)	.001	6(27.3)	8(38.1)	.001	8(38.1)	8(38.1)	<.001
Balance	BBS	Exp.	16(72.7)	18(90.0)	.053	16(72.7)	18(90.0)	.053	18(90.0)	18(90.0)	.005
		Control	6(27.3)	10(47.6)	.004	6(27.3)	10(47.6)	.004	10(47.6)	10(47.6)	.000
Activities of Daily	UPDRS-Part II	Exp.	9(40.9)	17(85.0)	.218	9(40.9)	18(90.0)	.479	17(85.0)	18(90.0)	.016
livings		Control	4(18.2)	8(38.1)	.012	4(18.2)	10(47.6)	.035	8(38.1)	10(47.6)	<.001

*UPDRS* Unified Parkinson’s Disease Rating Scale, *ABCS* Activities-specific Balance Confidence scale, *BBS* Berg balance scale, *Exp* Experimental

The findings showed robust improvements in nearly all outcome measures from baseline to all subsequent evaluations, with benefits lasting long after the experimental group's treatments ended.

### Within group differences

The means calculated for all the outcome measure in both groups are presented in Table 4 at 6-week, 12 weeks and 16 weeks duration. The results revealed a robust within-group increase across motor function, balance and balance confidence in the routine PT +VR+ MI group (*p*-value <.001) and improvements were maintained even at the follow-up while for ADL’s the difference was significant only when compared with post-intervention and follow-up assessments. For the Routine PT only group, the changes in motor function and balance were significant when comparison was made between 12^th^ and 16^th^ week assessments. Moreover, for the balance confidence and ADL’s, significant within- group improvements were observed with *p*<.001 (Table 4).

**Table 4:** With-in group comparison of mean scores of UPDRS-part II, III, BBS and ABCS

Outcome	Outcome Measures	Groups	Baseline	Assessment at 6^th^ Week	Assessment at 12^th^Week	Follow up at 16^th^ Week	Friedman Test	
			Mean ± SD	Mean± SD	Mean± SD	Mean± SD	χ^2^	p
Motor Function	UPDRS-Part III	Exp.	32.45±3.98	23.00±8.31	15.05±7.16	18.68±7.04	56.35	<.001
		Control	31.86±4.62	28.23±6.10	25.52±7.36	24.27±9.23	40.05	<.001
Balance confidence	ABCS	Exp.	59.26±5.87	73.55±4.45	81.01±6.14	78.60±5.76	60.39	<.001
		Control	59.34±8.89	66.39±7.80	71.83±8.25	68.65±7.33	53.87	<.001
Balance	BBS	Exp.	38.95±3.23	46.59±3.07	51.36±2.83	52.36±2.30	60.89	<.001
		Control	40.23±4.61	43.23±4.45	45.77±4.52	45.54±3.98	40.93	<.001
Activities of Daily livings	UPDRS-Part II	Exp.	22.00±4.64	17.14±4.36	13.07±4.005	12.85±4.050	53.40	<.001
		Control	21.51±3.89	20.03±3.80	18.00±4.19	16.54±4.61	50.49	<.001

*UPDRS* Unified Parkinson’s Disease Rating Scale, *ABCS* Activities-specific Balance Confidence scale, *BBS* Berg balance scale, *Exp* Experimental

## Discussion

Technological advancements in this era in the form of virtualization of rehabilitation have emerged. This technology is involved in patient assessment, treatment, and research [57]. Research has an important role to play in understanding the integration of VR and MI into rehabilitation plans of care. Therefore, continuous exposure on the part of researchers and patients is necessary for the determination of positive and adverse effects, if any [58]. In recent years, increasing knowledge of VR and MI has led to increased research in this aspect. Nevertheless, little is known about the combined effects of these two interventions on patients with PD. To the best of the authors’ knowledge, no study has yet examined the combined effects of VR and MI in combination with routine PT on PD. A well-designed protocol was used for these patients based on the clinical manifestations of PD in different clinical stages [32].

The current study, first of its kind, was conducted for the evaluation of the combined effects of routine PT, VR and MI. There is dearth of such studies in the literature. The combination therapy resulted in improvements in motor function, balance along with enhancing the balance confidence and all these together resulted in improved ADL performance. The results were not only evident at 6-weeks period but continued to progress at 12 –weeks with the addition of new patients and the results were then retained at follow-up as well. Significant results (both statistically and clinically) were evident by the analysis.

Statistically significant differences were reported in the motor function of the experimental group at the initial assessment performed at 6^th^ week after the continuation of treatment. These improvements continued to increase in the coming weeks (12^th^ week) and the results were even replicated after the discontinuation of the intervention (16^th^ week). The assessment performed on the patients using sections II and III of the UPDRS revealed a fact that has been reported in other studies on PD and other neurological conditions [59, 60]. A recently published systematic review categorically stated that VR is effective in improving motor function and ADLs [61]. Excluding the location of brain lesions, the motor imagination has had a reliable positive effect on motor-rehabilitation, either by increasing the treatment duration or by stimulating the neuromuscular pathways [62]. Similarly, Dual-task based gait or balance training using MI can enhance mobility and executive functions in patients with PD with a long-lasting effect [63]. Collectively, these two novel technologies might have created better outcomes in this recent study. In our study, the reason for the improved function might be that the PD patients tended to learn and acquire new skill patterns based on repetitions and memory recall and that the protocol was based around these notions. Although the exact mechanism is not well understood, the possibility exists that the exercise protocol may have enhanced external feedback, leading to improvements in motor skills and balance function. Overall, a positive effect of the treatment protocol was observed in the experimental group, greatly exceeding the therapeutic outcomes reported in other studies.

In interventional studies, determining the between-group difference is not simple. To ensure therapy efficacy, it is essential to identify clinically significant differences between study groups. It is vital to know how many patients in each group improved according to the MCID criteria for each outcome measure. At the 16-week evaluation, motor function improved in 95% of patients in the regular PT+VR+MI group (*p*-value=0.050), adopting 11 points MCID for UPDRS-III [55]. Assuming MCID for individual improvement was 5 points on BBS, 90% of individuals improved at follow-up (PT+VR+MI group, *p*=0.005) [56]. Similar gains were reported in the current trial, with 90% of participants exhibiting improvements in balance confidence and ADLs at the 16th week assessment [55].

People with PD have a significant proclivity for balance problems, which may result in falls [64]. The participants in the intervention group had balance problems and were treated according to a well-established protocol in this current study. The intervention group showed robust improvements in balance. As previously published studies show, virtual reality technology specifically developed to treat PD symptoms has favorable benefits, notably on balance and the risk of falls [10, 30, 65, 66]. Moreover, in the present study, the improvement was retained during the follow-up. Few studies in the literature have reported improvement in balance with the use of VR, but in most of those studies, progress was neither reported nor retained [57, 67, 68]. The study by Feng et al. recently advocated VR in the rehabilitation of PD patients for long-term effects [65]. Moshref and colleagues revealed that mental exercises have comparable and common neural processes to physical exercises at different phases of motion control. However, mental exercises are more effective than physical exercises since they produce no end movement [69]. This might be a result of the MI technique's added motor learning effect on the plan of care. Additionally, when VR and MI are coupled, the distinct components of motor learning attempts come into play, resulting in improved and additional impacts of these two unique treatments.

In the literature, there have been a lot of studies that show that people can learn motor skills in a virtual environment and then use them in the real world very well [70, 71]. This innovative technological advancement contributes to the impacts by assisting PD sufferers in determining positional sensation and movement direction in space. This effect is created via the collaboration of visual and somatosensory information in both stationary and dynamic body states [72, 73]. It is a well-established fact that performing body movements, whether static or dynamic, requires the establishment of a link between body alignment control, muscular tone, supporting surface, visual environment, and internal references to a point that can manifest as an athletic and balanced human [11]. Also, using technology in PD therapy allows for repeated movement practice, prompt performance feedback, and increased motivation [74]. A technology-based rehabilitation approach for this patient group has been shown to combine physical and cognitive processes (such attention and executive memory functions) and so activate brain circuits [11]. Moreover, Kobelt and colleagues found that MI may cause subliminal EMG activity in targeted muscles. The consequences may differ from person to person [75]. Summarizing these mechanisms, it can be claimed that VR and MI, in conjunction with routine PT, may improve motor functioning and balance, resulting in increased independence of PD patients in executing ADLs, as shown by the findings of the current study.

The combined use of MI or VR has been shown to be superior to PT when used alone, according to the studies. According to one study, combining Nintendo Wii and traditional exercise improves gait, mobility, and overall quality of life [8]. Similarly, another study compared Tele Wii and SIBT and concluded that Tele Wii had superior effects [11]. Balance and gait measures improved in the VR group after three and twelve months of follow-up, but not in the control therapy group, as Shen et al. reported [76]. Patients in virtual environments perform tasks repeatedly, gain feedback about performance, and enhance motivation, which is critical in patients with PD [74]. When used in conjunction with physical therapy, MI improves patients' motivation, concentration, and attention when compared to PT treatment alone in patients with neurological conditions [77]. PD patients experience a reduction in bradykinesia. There is a possibility that MI will play an important role in the cognitive strategies provided to these patients [78]. Moreover, It has been shown that combining MI with dual-task gait/balance training improves dual-task mobility and balance in PD patients with postural instability and gait disorders [63].

Although no previously published study has addressed the combined effects of VR and MI therapy in patients with PD, these techniques have been used individually on other neurological deficits. Several studies have reported improvements in patients with other neurological conditions, but the outcomes in these individuals appear to be greater across different training approaches [79]. In the current study, this effect can be explained by the increased demands placed on implicit and explicit memory systems. An additional benefit of this combination protocol is the performance of the original pattern of movement compared with other technological advancements. The movement impairments and constraints in these patients are due to unusual movement timings and coordination deficits. Muscle weakness and decreased range of motion have a secondary role in movement limitations, in contrast to other neurological abnormalities. Coordination assessments are not performed on a routine basis among PD patients, and coordination deficits cannot be overcome by manual techniques in a better way. The results of these deficits are inadequate movement and abnormal movement patterns [80]. VR and MI in a blended approach primarily help normalize the pattern of movement initiation and completion. These innovative techniques also assist patients in modifying ineffective movements and actively avoiding these when a need arises. PD patients have a limited ability to learn new tasks and use new movement patterns in ADLs.

In the current study, significant differences were observed after 18 sessions of therapy in the intervention group, with each session lasting for 60 min. A previous study recommended at least eight sessions with 40-min therapy for the results to be statistically significant [81]. This aspect should be explored further in future research.

## Conclusion

This study found that combining VR and MI techniques with PT treatment produced a clinically significant effect on motor function, balance, and ADLs, which lasted longer in PD patients compared with PT treatment alone.

### Limitations

Due to the unique nature of VR and MI technological interventions and strategies, the patients in the experimental group could not be blinded. This may be responsible for the deviations in the subjective data in the final results. To incorporate this technological advancement more often in the PT plan of care, there is a need to gather data on the cost-effectiveness of VR and MI training compared with routine PT, as only one study has so far reported the cost of VR equipment. Objectively oriented kinematic readings should be obtained in future studies to enable a better understanding of the sub-sections for improvement. Moreover, it is difficult to ensure that patients will imagine the movements and that they will use this imagination in the kinesthetic modality and in the number of repetitions proposed.

## Data Availability

The data generated or analyzed during this study are presented in this article and for further enquiries can be directed to the corresponding author.
